# Interpersonal violence and the prediction of short-term risk of repeat suicide attempt

**DOI:** 10.1038/srep36892

**Published:** 2016-11-14

**Authors:** Axel Haglund, Åsa U. Lindh, Henrik Lysell, Ellinor Salander Renberg, Jussi Jokinen, Margda Waern, Bo Runeson

**Affiliations:** 1 Department of Clinical Neuroscience, Centre for Psychiatry Research, Karolinska Institutet, Stockholm, Sweden; 2 Department of Clinical Science, University of Umeå, Sweden; 3 Department of Psychiatry and Neurochemistry, University of Göteborg, Sweden

## Abstract

In this multi-center cohort study, suicide attempters presenting to hospital (N = 355, 63% women) were interviewed using the Karolinska Interpersonal Violence Scale (KIVS) and followed-up by medical record review. Main outcome was non-fatal or fatal repeat suicide attempt within six months. Also, repeat attempt using a violent method was used as an additional outcome in separate analyses. Data were analyzed for the total group and for men and women separately. Repeat attempts were observed within six months in 78 persons (22%) and 21 (6%) of these used a violent method. KIVS total score of 6 or more was associated with repeat suicide attempt within six months (OR = 1.81, CI 1.08–3.02) and predicted new attempts with a sensitivity of 62% and a specificity of 53%. A three-fold increase in odds ratio was observed for repeat attempt using a violent method (OR = 3.40, CI 1.22–9.49). An association between exposure to violence in adulthood and violent reattempt was seen in women (OR = 1.38, CI 1.06–1.82). The overall conclusions are that information about interpersonal violence may help predict short-term risk for repeat suicide attempt, and that structured assessment of interpersonal violence may be of value in risk assessment after attempted suicide.

Suicide and suicide attempts are major public health concerns worldwide^1^. Essential for prevention is to facilitate the identification of patients with high risk for suicidal behavior in the short-term perspective after a health care visit^2^. A suicide attempt is the strongest known risk factor for later suicide^3^,^4^ and approximately half of all people who die by suicide have made at least one previous attempt^5^,^6^. In the year prior to their death from suicide, 15–20% visited a hospital in connection with a suicide attempt in a large study based in the UK^7^.

Suicide attempters presenting to hospital constitute a high-risk group for repeat suicide attempts. Young age and female gender influence the risk of repetition^8^,^9^,^10^,^11^. Coexisting severe psychiatric disorder and violent method used at an initial attempt indicate higher risk of a subsequent suicide^3^,^12^,^13^. A repeat attempt further elevates the risk of suicide mortality^14^,^15^. However, most studies of risk factors among suicide attempters have used a long-term follow-up (from years to decades), and therefore provide limited guidance in the shorter time perspective. For clinical management, the risk of repeat attempt in a time frame of weeks and months is more relevant for decisions concerning the immediate care^16^. Further research on factors related to short-term risk among suicide attempters is needed to guide preventive efforts^2^,^16^.

Violent methods such as using firearms, hanging and jumping from a height are associated with higher immediate lethality of an attempt^17^ as well as elevated risk of future suicide^12^,^13^. Further, violent methods are less common than poisonings and have been shown to be associated with higher levels of reported lifetime aggression^18^. Thus, identification of factors associated with violent suicide attempts may improve risk assessments.

An early traumatic life event is a known risk factor for later suicide 4, and exposure to violence in childhood is associated with later suicidal behavior^19^. Also, bullying victimization increases the risk for suicidal behavior as an adult, even after adjustment for psychiatric comorbidity^20^,^21^. A relationship between exposure to domestic violence and risk for suicide attempts has also been reported^22^. However, not only the victim of violence, but also the perpetrator, seems to have an increased risk for suicidal behavior^4^. Mutual pathways to aggressive and suicidal behavior have been suggested to involve traits of impulsivity and aggression^4^,^23^. High levels of aggression have been found among individuals who died by suicide when compared with psychiatric control subjects^24^.

Increased risk of suicide has been observed among suicide attempters with an experience of interpersonal violence^25^. The Karolinska Interpersonal Violence Scale (KIVS) is a short and useful tool for gathering information about a patient’s experiences of interpersonal violence as a child and as an adult, and both of exposure and expression of violence. The severity of the violence is graded using preset definitions (Appendix 1). Exposure to violence as a child and expression of violence as an adult measured with KIVS predicted suicide in a study with a follow-up period of 4–15 years^25^. In another study based on the same cohort, the predictive value of a combination of the KIVS and the Suicide Intent Scale (SIS) was analyzed, and the positive predictive value was enhanced further^26^. The scores on KIVS and SIS did not correlate with each other, indicating that the scales measure different aspects contributing to the risk of suicide in a long-term perspective. However, whether information about interpersonal violence can help predict suicide attempts in a short-term perspective has not been studied before. Therefore, in this clinical cohort study of suicide attempters, we aimed to evaluate the ability of the Karolinska Interpersonal Violence Scale to predict a repeat suicide attempt (non-fatal or fatal) within a short-term follow-up period of 6 months.

Our research questions were:

1. Can information about interpersonal violence, measured with KIVS, predict repeat suicide attempt within six months?
2. Are high scores on KIVS associated with repeat suicide attempt using a violent method?
3. Does the KIVS’s ability to predict suicide attempts differ according to gender?

## Method

### Study settings and population

This study is the first to use data from a multicenter study conducted in Sweden since 2012 at three psychiatric departments: at St. Göran’s Hospital in Stockholm, at Sahlgrenska University Hospital in Gothenburg and at Umeå University Hospital. Patients aged 18 and above presenting to hospital within a week after an act of deliberate self-harm with or without suicide intent were considered for participation. To enable follow-up through medical records, the inclusion criteria also included having a Swedish personal identity number, and being a resident of the catchment area of the specific hospital. Patients who were deemed unable to take part in the interview, either due to lacking language skills or due to acute symptoms interfering with verbal communication, were not asked to participate in the study. This included persons who were too confused, aggressive, psychotic or demented. Eligible patients were asked to participate and all participants gave written informed consent. Structured interviews were conducted within days after presenting to the hospital. Specially trained mental health care professionals conducted all interviews during daytime from April 25, 2012 to April 28, 2014.

### Scorings and outcomes

The interview included the KIVS as well as a set of other instruments not analyzed in the present study. The KIVS consists of four subscales measuring expressions of violence and exposure to violence as a child (age 6–14), and expressions of violence and exposure to violence as an adult (age 15 and above). The two subscales capturing interpersonal violence as a child (age 6–14) also include experiences of bullying and bullying victimization. All subscales are scored from 0 to 5, with 0 corresponding to no violence at all and 5 to severe forms of interpersonal violence. Hence, the maximum total score on the KIVS is 20 points. In addition to the five continuous variables based on the total score and the four subscales of the KIVS, a dichotomized variable was created based on the total scores split at the median (0–5 vs. 6 or more). The reason for adding this binary variable in the analyses is to investigate a cut-off that possibly could be of pragmatic clinical use. This precise cut-off was chosen mainly because the score of 6 was both the mean and the median score of the suicide attempters in the previous cohort studied by Jokinen and coworkers 2010, but also because this score implies that at least two subscales have generated the score, not merely one. Also, the score of at least one subscale needs to be 2 or higher. For instance, a person with a total score of 6 or more has at least been involved in physical fights or has been bullied as a child, or has been either the perpetrator or victim of intimate partner violence as an adult. The full scale is presented in Appendix 1. The KIVS has previously been validated against the Buss–Durkee Hostility Inventory (BDHI), the “Urge to act out hostility” item from the Hostility and Direction of Hostility Questionnaire (HDHQ), and the Early Experiences Questionnaire (EEQ)^25^. The inter-rater reliability in the same study varied from r = 0.91 to r = 0.95 in the four subscales^25^. To be able to describe the cohort in diagnostic terms, the interview also included a synoptic diagnostic screening using the Mini International Neuropsychiatric Interview (M.I.N.I.)^27^.

Outcome was a new fatal or non-fatal suicide attempt within six months after the index attempt. We used the definition of actual suicide attempt used in the C-SSRS: a potentially self-injurious act committed with at least some wish to die, as a result of the act^28^. In additional analyses we used suicide attempt with a violent method as the outcome, defining violent method as all methods except poisoning (i.e. cutting, hanging, using shotgun, jumping from a height etc.). This distinction between violent method and poisoning is established in previous research^17^,^29^. It is also supported by research showing that the risk of subsequent suicide is significantly lower after poisoning when compared with other methods^12^,^13^. Information regarding outcome was extracted from the patient’s electronic medical records.

### Statistical analyses

T-tests (2-tailed) were used to detect significant gender differences in mean age, and chi-square tests to determine gender differences in the results on the KIVS and the prevalence of diagnoses. Logistic regression analysis was used to calculate odds ratios for repeat suicide attempt. The four subscales and the total score were analysed as continuous variables. The odds ratios represent how risk changes with a one-step elevation on the scores. The variable “KIVS total score 6 or more” was analysed as a dichotomous variable. No adjustments have been made in the logistic regression models. Receiver operating characteristics (ROC) curves were used to further evaluate predictive properties of the KIVS subscales and the total score. All gender-specific results were made through separated analyses for men and women. All statistical analyses were made with SPSS Version 22.0^30^.

### Ethical statement

Ethical approval was obtained for this study from the Gothenburg Regional Ethics Committee (589–10). The methods used in this study were in accordance with the ethical approval. Written informed consent was obtained from all included subjects.

## Results

### Participants

In total 665 self-harming patients were eligible for inclusion and 452 agreed to participate (68.0%). For the purpose of this paper, 82 persons without suicidal intention at the index self-harm act as defined by the Columbia Suicide Severity Rating Scale^28^ were excluded from the analyses, as were those lacking KIVS data (n = 15). The current study is thus based on data for 355 suicide attempters (133 men and 222 women), with a mean age of 40 years (range 18 to 92 years, median 35). The women were younger than the men (mean age 37 years vs. 45, p < 0.001), and women predominated in age groups up to 44 years (Table 1). According to the results of the M.I.N.I.-interviews, a majority (60%) of the participants met criteria for ongoing major depression. An ongoing episode of bipolar disorder was reported in 15% of the participants. Anxiety disorders were common (72%), and ongoing psychotic syndrome was found in 6%. The only significant gender differences observed were found in the diagnostic categories of ongoing substance use disorder (47% of male participants and 31% of female, p = 0.004), and ongoing eating disorder which was reported by 13% of the female participants and 3% of the men (p = 0.003).

### Risk of repeat suicide attempt

In the cohort of 355 suicide attempters, 78 (22.0%) made a fatal or non-fatal repeat suicide attempt within six months (50 women, 28 men) and five of these were suicides (three women, two men). Violent methods were used in four of these suicides (one man used poisoning as method). In all, fatal or non-fatal repeat attempt with violent method was recorded for 21 participants (5.9%). Sixteen were noted for women (7.2% of all women) and five for men (3.8%).

### KIVS score

The mean total KIVS score was 6.0 with no gender difference (men 6.1, women 6.0, p = 0.189). Gender differences were observed with higher scores for men in the two subscales covering expression of violence, and higher scores for women in the two subscales covering exposure to violence. There was no significant gender difference in the total scores (Table 2).

### KIVS score in relation to risk of repeat suicide attempt

In bivariate logistic regression, having a KIVS total score of 6 or more was associated with an increased risk for repeat suicide attempt within six months (Odds Ratio = 1.81, CI 1.08–3.02). The subscale “exposure to violence as an adult” was associated with an increased risk for repeat suicide attempt within six months (OR = 1.14, CI 1.01–1.29) (Table 3).

**Figure 1:**
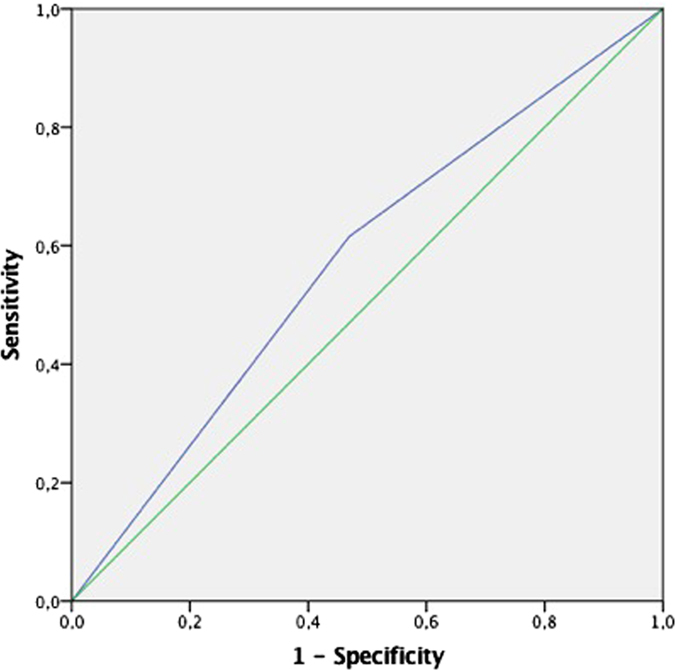
ROC-curve for the KIVS subscale “KIVS total score of 6 or more” predicting fatal or non-fatal suicide attempt within six months, men and women combined.

The significant subscales in the logistic regression analysis were tested further using ROC curves. The dichotomous variable “KIVS total score of 6 or more” generated a ROC curve with an Area Under the Curve (AUC) of 0.57 (p = 0.049) (Fig. 1). A result of 6 or more points on the KIVS predicted a repeat suicide attempt with a sensitivity of 62% and a specificity of 53%. A KIVS total score of 6 or more was also associated with an increased risk for a new attempt using a violent method (OR = 3.40, CI 1.22–9.49) (Table 4). The subscale “exposure to violence as an adult” could not significantly predict a new suicide attempt within six months when analysing its ROC curve (AUC = 0.57, p = 0.065) (ROC curve not shown).

### Gender-specific analyses

Neither the total score of KIVS nor any of the subscales were significantly associated with repeat suicide attempt within six months in the gender-specific analysis. However, gender differences unfolded when analyzing the outcome repeat attempt using violent method. In women the dichotomous variable KIVS total score of 6 or more was associated with an almost fivefold increase in risk (Table 4). Also, higher results on the subscale “exposure to violence as an adult” increased the risk of repeat suicide attempt using a violent method within six months among women (OR = 1.38, CI 1.05–1.81, p = 0.020). No associations were observed in men.

**Figure 2:**
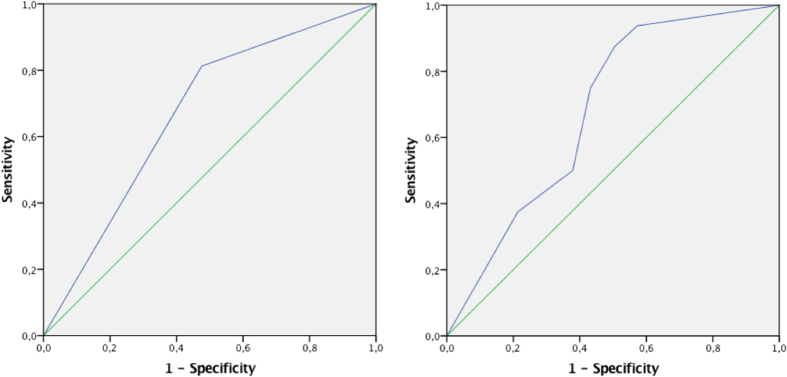
ROC-curves for the subscales (**A**) “KIVS total score of 6 or more”, and (**B**) “Exposure to violence as an adult” predicting fatal or non-fatal suicide attempt with a violent method within six months, women only.

The ROC curve for the dichotomous variable of KIVS total score of 6 or more used as a test to predict repeat suicide attempt using a violent method among women showed an AUC = 0.67 (p = 0.025) (Fig. 2A) and a sensitivity of 81% and a specificity of 52%.

Using the subscale “exposure to violence as an adult” as a test to predict a new suicide attempt using a violent method within six months among women generated a ROC curve with an AUC of 0.68 (p = 0.016) with an optimal cut-off point at 3 generating a sensitivity of 75% and a specificity of 57% (Fig. 2B).

## Discussion

To our knowledge, this is the first study to link a certain degree of reported interpersonal violence with an elevated short-term risk of repetition of suicidal behavior. Repetition has in itself been found to double the risk for eventual suicide^15^, making our finding relevant from a suicide prevention perspective. We found that scoring 6 or more on the Karolinska Interpersonal Violence Scale was associated with repeat suicide attempt within six months. To reach a score of 6 or more on the KIVS implicates that at least two of the subscales are involved, assuring that not only one single occasion of interpersonal violence explains the total score. It also means that more severe forms of interpersonal violence must have occurred. Still, a score of 6 or more can be reached in many different ways, since the scale captures a broad variety of experiences.

The overall predictive value of the scale should not be overstated based on our results. Both the sensitivity (62%) and the specificity (53%) to detect repeat attempts are unsatisfactory, although not far from the predictive properties of other suicide risk assessment tools previously evaluated in the literature^16^. Nevertheless, an aspect that makes our findings interesting is that the KIVS, contrary to most other suicide risk assessment tools, does not include any questions directly about psychiatric symptoms or suicidal ideation/behavior. It illuminates other relevant experiences of interpersonal violence that may have been previously overlooked in suicide risk assessments.

Experiences of interpersonal violence were common in the cohort. The mean total KIVS score of 6.0 was consistent with that reported in a long-term Swedish follow-up study of suicide attempters^25^. The latter study also included a healthy control group with a mean total score of 2.9, showing that suicide attempters experience interpersonal violence to a larger extent^25^. Findings from cross-sectional studies of alcohol dependent^31^ and borderline^32^ patients support the notion that information about interpersonal violence have relevance for prediction of suicidal behavior in different clinical samples.

We found that being a victim of violence as an adult was associated with repeated suicidal behavior. None of the other three subscales were in themselves significantly associated with the outcome. This is in contrast with the earlier longitudinal study^25^ where the subscales covering exposure to violence as a child and expression of violence as an adult were most strongly linked with suicide. This is probably due to differences in study design since the present study use a different outcome, including both non-fatal and fatal attempts, and a shorter follow-up time. The results may indicate that different kinds of experiences of interpersonal violence affect subsequent risk for attempted suicide and completed suicide differently. For an adult, to have been a victim of violence in childhood could be considered as a distal risk factor, whereas victimization in adulthood is more of an acute stressor associated with suicidal behavior in a shorter time perspective. Also, the more impulsive nature of suicide attempts than of completed suicide^33^ could be a reason that a proximal stressor, the experience of being exposed to violence more recently in adulthood, is of particular relevance to the outcome of suicide attempts in the present study.

The answer to our main research question is that information about interpersonal violence may contribute to the prediction of new suicide attempts in a short time perspective.

Our second research question concerned the prediction of violent reattempts. Having a total score of 6 or more on KIVS elevated the risk for a new violent attempt significantly and with a higher point estimate (OR = 3.4) than for repeat attempt regardless of method (OR = 1.8). This may indicate that the violent attempters as a group is closer to the suicide phenotype, previously shown to be associated with higher scores on the KIVS^25^.

The third research question, about gender differences, could not be unequivocally answered, since the confidence intervals of the risk estimates in the gender-stratified analyses were too broad to separate the odds ratios for men and women. However, a possible gender difference was observed when analyzing risk for violent reattempt. The finding that being a victim of violence as an adult could predict violent suicide attempts among female suicide attempters harmonize with previous research showing that domestic violence against women is associated with suicide attempts^34^. For men we found no significant association, but the number of men with violent reattempts was small, limiting the statistical power. Violent methods are associated with higher immediate lethality^17^ and elevated risk of later suicide^12^,^13^, probably to a certain extent because it indicates a stronger suicidal intention. In large epidemiological studies of suicide attempters, women tend to choose violent methods more seldom than men^12^,^13^, in contrast with our present result. An important methodological difference that partly may explain this is that we in our clinical sample excluded self-harmers without suicide intent, something that cannot be done in larger epidemiological studies based on data relying on ICD-10 codes, which do not differentiate between non-suicidal self-harm and suicide attempt. However, this distinction is relevant since the choice of method in a self-harm act most likely is linked with the intention of the act. Another possible reason for the observed gender difference is that interpersonal violence experienced by younger women may have a slightly different impact on their development of suicidal behavior than in men, perhaps rendering the latter more likely to use violent methods. In line with the interpersonal theory by Joiner^35^, the exposure of violence may have desensitized these women to experiencing physical pain, making them more capable of violent self-injurious behavior.

A recent Swedish population study showed a link between depression and violent crime^36^. The authors conclude that assessment of risk of violence, which to a large extent depends on information about previous experiences of interpersonal violence, should be considered routinely for certain subgroups of patients with depression, including those who have engaged in self-harm. The results from our present study support a similar notion that questions about interpersonal violence also could be relevant from a suicide risk assessment perspective, and for this reason should be used more often in certain clinical situations such as in the aftermath of a suicide attempt. However, despite the significant association with the outcome, the KIVS provides limited sensitivity and specificity in predicting repeat attempts on its own, and should only be considered as a complement to regular risk assessment.

Also, further research is needed to disentangle the associations and causal pathways between interpersonal violence and suicidal behavior, to enable even better prediction and tailor-made preventive interventions.

### Strengths and limitations

The large sample size enabled sufficient power to analyze short-term risk for a repeat suicide attempt, and even to some extent, gender-specific analyses. However, the study sample was too small to also allow further division in subgroups according to age and diagnosis, hence these variables could only be used to describe the cohort. Also, the number of suicide deaths was too small to enable separate analyses. A larger sample size is needed to determine whether findings in the present study may also pertain to suicide mortality.

All participants included in the present study acknowledged suicidal intention at the index attempt, which makes our findings easier to interpret clinically compared with register-based studies dependent on ICD-codes that also include acts of deliberate self-harm without suicidal intention. In this study we rely on self-reported information about interpersonal violence. There is risk of recall bias when asking delicate questions shortly after a traumatic event such as a suicide attempt. Also, patients who were too aggressive to participate were not included, generating a selection bias towards a perhaps less aggressive patient population. These limitations should be considered when generalizing our results to other patient populations.

Information about the studied outcome was extracted from the patient’s electronic medical records. Suicide attempts that were not reported in the medical records are lacking. This introduces a bias that probably favors more severe forms of suicidal behavior with higher probability of being reported in medical records. This is important to remember when interpreting our results.

Finally and importantly, the significant findings reported in this study have p-values not far from 0.05. This in itself means that the validity of the results needs to be confirmed in future studies. Furthermore, for a clinician, the predictive value of this scale, as with all other scientifically evaluated suicide risk assessment tools, is unsatisfactory, and we can merely propose that aspects of interpersonal violence may be included in the clinical assessment of patients who have attempted suicide.

## Conclusion

In this cohort study of 355 suicide attempters, self-reported history of interpersonal violence was associated with elevated risk for a new fatal or non-fatal attempt within a clinically relevant follow-up period of six months. Questions regarding interpersonal violence may help to inform clinical assessments in the aftermath of a suicide attempt.

## Additional Information

**How to cite this article**: Haglund, A. *et al.* Interpersonal violence and the prediction of short-term risk of repeat suicide attempt. *Sci. Rep.* **6**, 36892; doi: 10.1038/srep36892 (2016).

**Publisher’s note**: Springer Nature remains neutral with regard to jurisdictional claims in published maps and institutional affiliations.

## Supplementary Material

Supplementary Information

## Figures and Tables

**Table 1:** Total cohort of 355 suicide attempters, by age and gender.

Age	All		Men		Women		P- value**
	N	%*	N	%*	N	%*	
**18–24**	93	26.2	23	17.3	70	31.5	0.001
**25–34**	83	23.4	30	22.6	53	23.9	0.012
**35–44**	50	14.1	15	11.3	35	15.8	0.005
**45–54**	54	15.2	27	20.3	27	12.2	1.000
**55–64**	34	9.6	18	13.5	16	7.2	0.732
**>65**	41	11.5	20	15.0	21	9.4	0.876
**Total**	**355**	100	**133**	100	**222**	100	<0.001

^*^Percentage of Total N.

^**^P-value for gender differences within age group using chi-square test.

**Table 2:** All results on the Karolinska Interpersonal Violence Scale (N = 355).

	Gender	Mean	P-value*
**Expression of violence as a child**	Men	0.83	0.036
	Women	0.58	
	Both	0.67	
**Expression of violence as an adult**	Men	1.02	0.034
	Women	0.64	
	Both	0.78	
**Exposure to violence as a child**	Men	2.13	0.004
	Women	2.54	
	Both	2.39	
**Exposure to violence as an adult**	Men	2.13	0.039
	Women	2.20	
	Both	2.17	
**KIVS total score**	Men	6.11	0.189
	Women	5.96	
	Both	6.02	
**KIVS total score 6 or more**	Men	0.50	0.945
	Women	0.50	
	Both	0.50	

Men (N = 133) and women (N = 222) shown separate and together.

^*^Significance of gender difference (Pearson chi-square test).

**Table 3:** Bivariate logistic regression generated odds ratios (OR) for one-step elevation in score at the Karolinska Interpersonal Violence Scale, and all of the subscales, and the outcome repeat suicide attempt within six months, men and women shown separate and both together.

	Gender	OR	C.I. lower	C.I. upper	P-value
**Expression of violence as a child**	Men	1.02	0.74	1.42	0.886
	Women	1.09	0.85	1.40	0.504
	Both	1.06	0.87	1.29	0.564
**Expression of violence as an adult**	Men	1.21	0.91	1.60	0.191
	Women	0.90	0.66	1.24	0.519
	Both	1.05	0.85	1.28	0.675
**Exposure to violence as a child**	Men	1.10	0.88	1.38	0.397
	Women	1.10	0.92	1.31	0.297
	Both	1.10	0.96	1.26	0.171
**Exposure to violence as an adult**	Men	1.15	0.92	1.42	0.212
	Women	1.13	0.97	1.32	0.107
	Both	1.14	1.00	1.29	0.041*
**KIVS total score**	Men	1.06	0.97	1.16	0.191
	Women	1.05	0.97	1.12	0.212
	Both	1.05	0.99	1.11	0.074
**KIVS total score 6 or more**	Men	2.06	0.87	4.88	0.101
	Women	1.69	0.89	3.20	0.110
	Both	1.81	1.08	3.02	0.024*

No adjustments were made.

^*^Significant increase in risk (p < 0.05).

**Table 4:** Bivariate logistic regression generated odds ratios (OR) for one-step elevation in score at the Karolinska Interpersonal Violence Scale, and all of the subscales, and the outcome repeat suicide attempt using violent method within six months, men and women shown separate and both together.

	Gender	OR	C.I. lower	C.I. upper	P-value
**Expression of violence as a child**	Men	0.83	0.35	1.99	0.682
	Women	1.12	0.77	1.63	0.556
	Both	1.03	0.72	1.46	0.873
**Expression of violence as an adult**	Men	0.88	0.43	1.81	0.724
	Women	1.04	0.65	1.67	0.863
	Both	0.95	0.65	1.39	0.786
**Exposure to violence as a child**	Men	1.02	0.63	1.65	0.929
	Women	1.34	0.98	1.84	0.068
	Both	1.26	0.97	1.62	0.077
**Exposure to violence as an adult**	Men	0.75	0.43	1.29	0.292
	Women	1.38	1.05	1.81	0.020*
	Both	1.21	0.97	1.51	0.094
**KIVS total score**	Men	0.93	0.75	1.15	0.514
	Women	1.13	1.01	1.26	0.032*
	Both	1.08	0.98	1.18	0.133
**KIVS total score 6 or more**	Men	1.50	0.24	9.28	0.663
	Women	4.78	1.32	17.26	0.017*
	Both	3.40	1.22	9.49	0.020*

No adjustments were made.

^*^Significant increase in risk (p < 0.05).
